# Systemic inflammation and prognosis in patients with *RAS* / *BRAF* wild‐type metastatic colorectal cancer receiving anti‐EGFR therapy: A real‐world cohort study

**DOI:** 10.1111/codi.70639

**Published:** 2026-09-25

**Authors:** Jordan W. Appleyard, Faye Elliott, Nancy L. Sapanara, Liping Zhang, Uday Kurkure, Jenny F. Seligmann, Nicholas P. West, Kandavel Shanmugam, Philip Quirke, Christopher J. M. Williams, the N6 Collaboration

**Affiliations:** ^1^ Division of Oncology, Leeds Institute of Medical Research University of Leeds Leeds UK; ^2^ Division of Haematology and Immunology, Leeds Institute of Medical Research University of Leeds Leeds UK; ^3^ Clinical Development & Medical Affairs Roche Diagnostics Solutions Tucson Arizona USA; ^4^ Computational Science and Informatics Roche Diagnostics Solutions Santa Clara California USA; ^5^ Division of Pathology and Data Analytics, Leeds Institute of Medical Research University of Leeds Leeds UK

**Keywords:** amphiregulin, anti‐EGFR, cetuximab, epiregulin, metastatic colorectal cancer, neutrophil‐to‐lymphocyte ratio, panitumumab, platelet‐to‐lymphocyte ratio, systemic immune‐inflammation index

## Abstract

**Aim:**

Colorectal cancer outcomes are heterogeneous, so greater prognostic discrimination is needed. In this retrospective real‐world analysis, we assessed the prognostic effect of markers of systemic inflammation in a cohort of patients with *RAS/BRAF* wild‐type metastatic colorectal cancer (mCRC) who received anti‐EGFR therapy during routine care. We further examined the association between these markers and the known predictive biomarkers of anti‐EGFR efficacy, amphiregulin (AREG) and epiregulin (EREG).

**Method:**

The prognostic effects of the neutrophil‐to‐lymphocyte ratio (NLR), platelet‐to‐lymphocyte ratio (PLR) and systemic immune‐inflammation index (SII) were tested. The cohort was randomly partitioned into training and validation sets (1:1). Cutoff values were defined in the training set, with analyses repeated in the validation set. The primary endpoint was overall survival (OS). Secondary endpoints were progression‐free survival (PFS), objective response rate and disease control rate.

**Results:**

374 patients with *RAS* and *BRAF* wild‐type mCRC who received treatment with cetuximab or panitumumab across eight UK centres were included. NLR (≤ 4 vs. > 4) and PLR (≤ 200 vs. > 200) were independently prognostic for both OS and PFS. SII (≤ 900 vs. > 900) gave the strongest prognostic signal (OS: adjusted HR 1.63 [1.26–2.11], *p* = 0.0002 and PFS: adjusted HR 1.62 [1.26–2.08], *p* = 0.0001). There were no associations between NLR or PLR with AREG or EREG, suggesting anti‐EGFR benefit in AREG/EREG‐high tumours is not related to systemic inflammation.

**Conclusion:**

Routinely assessed markers of systemic inflammation are independently prognostic in patients with mCRC receiving anti‐EGFR therapy and may serve as useful adjuncts in informing discussions with patients about prognosis.

**What Does This Paper Add to the Literature?**

Outcomes from anti‐EGFR therapy in *RAS*/*BRAF* wild‐type mCRC are heterogeneous. NLR and PLR are cheap, simple prognostic indicators. NLR and PLR had strong and independent prognostic effects, but do not explain the predictive effects of EGFR ligands, AREG and EREG. SII combines information from NLR and PLR, enhancing prognostic precision.

## INTRODUCTION

Colorectal cancer (CRC) is a biologically heterogeneous disease with different biomarkers, treatments and outcomes [1]. Markers of systemic inflammation have shown broad utility across a variety of tumour types [2, 3, 4] and non‐cancer related conditions such as cardiovascular diseases [5]. Whether and how these additional biomarkers can be used to improve patient selection for systemic anti‐cancer therapy (SACT) in more specific clinical scenarios is not yet defined.

An extended panel of *RAS* mutations (*KRAS*/*NRAS* codons 12, 13, 59, 61, 117 and 146) predict insensitivity to anti‐EGFR agents (cetuximab and panitumumab), while *BRAF* codon 600 mutations are probable negative predictive biomarkers [6, 7, 8, 9]. This explains why their status is routinely reported in clinical practice, with anti‐EGFR treatment restricted to patients with *RAS*/*BRAF* wild‐type disease in the first‐line setting [10]. *RAS*/*BRAF* wild‐type disease has a favourable prognosis in comparison with *RAS*/*BRAF* mutant disease in localised CRC that is proficient mismatch repair/microsatellite stable [11], as well as metastatic colorectal cancer (mCRC) [12]. However, prognosis still varies considerably amongst the wild‐type subset, emphasising the need for additional biomarkers to enhance prognostic discrimination.

Inflammation is an established hallmark of cancer that can promote tumorigenesis [13]. Neutrophil, lymphocyte and platelet counts can be abnormal through different mechanisms in patients with advanced cancer. Neutrophilia occurs because of tumoral production of cytokines that stimulate granulopoiesis [14]. It also occurs because of elevated numbers of myeloid‐derived suppressor cells. These immature cells produce immunosuppression that impacts the adaptive immune response and facilitates metastasis [15]. Neutrophilia is often accompanied by a relative lymphocytopenia [16]. Thrombocytosis is secondary to increased interleukin‐6, which stimulates hepatic thrombopoietin [17]. Not only are platelets proangiogenic [18], but they contribute to metastasis by shielding circulating tumour cells and protecting them from shear stress [19].

To clinically capture these biochemical changes, two prognostic measures have been proposed: the neutrophil‐to‐lymphocyte ratio (NLR) and platelet‐to‐lymphocyte ratio (PLR) [20, 21]. While these measures have shown broad utility across a variety of tumour types [2, 3, 4], the strength of their prognostic effect in *RAS*/*BRAF* wild‐type mCRC has not been specifically assessed—a factor that has limited their uptake in routine clinical practice. Moreover, while a variety of cutoff values have been proposed to usefully dichotomise patients into superior and inferior prognostic groups, these have never been tested in this setting specifically.

As described, different mechanisms are proposed by which elevated NLR or PLR might confer an inferior prognosis. Therefore, combining information from neutrophils, lymphocytes and platelets might provide greater prognostic precision. One proposed method for this is the systemic immune‐inflammation index (SII) that has been shown to have a prognostic effect in hepatocellular carcinoma [22].

High tumour expression of the EGFR ligands, amphiregulin (AREG) and epiregulin (EREG), indicates EGFR pathway dependence and anti‐EGFR sensitivity in CRC [6, 23, 24]. Whether there is any association between AREG/EREG expression and systemic inflammation—that could in part explain the favourable prognosis of patients with AREG/EREG‐high disease undergoing anti‐EGFR therapy [25]—has also not been previously explored.

Here, we examine the prognostic effect of NLR, PLR and SII in patients with *RA*S/*BRAF* wild‐type mCRC who received either cetuximab or panitumumab during routine care at one of eight UK cancer centres. It was hypothesised that high NLR and PLR would be associated with an inferior prognosis and that a combined approach like SII would yield a stronger association. In a descriptive analysis, we also assessed the relationship between markers of systemic inflammation and AREG/EREG immunohistochemistry (IHC).

## METHOD

### Patient recruitment

Eligibility criteria for recruitment to this patient cohort (NCT03986541) have been previously described [25]. Briefly, participants were aged 18 years or older with *RAS* wild‐type, histologically proven advanced colorectal adenocarcinoma (either inoperable metastatic disease at diagnosis or inoperable recurrent disease) who had received or were receiving cetuximab or panitumumab as part of routine care at one of eight UK cancer centres.

A total of 541 patients were recruited, and 449 patients were included following central extended *RAS* testing (codons 12, 13, 59, 61, 117 and 146 for both *KRAS* and *NRAS*) by next‐generation sequencing [25].

Ethical approval was granted as previously reported [25] and was appropriate for the purpose of conducting further exploratory analyses. The study was performed in accordance with the Declaration of Helsinki.

### Outcome measures

The primary endpoint in this prospectively planned, retrospective analysis was overall survival (OS; time from commencement of anti‐EGFR treatment to death from any cause). Secondary endpoints were progression‐free survival (PFS; time from commencement of anti‐EGFR therapy until radiological or clinical evidence of disease progression, as determined using local radiologist's interpretation of response, or death from any cause, whichever was sooner), locally assessed objective response rate (ORR; complete or partial response on first follow‐up imaging) and disease control rate (DCR; stable disease, complete or partial response on first follow‐up imaging).

### Measurement of biomarkers

Blood neutrophil count (10^9^/L), lymphocyte count (10^9^/L) and platelet count (10^9^/L) were recorded where these had been measured as part of routine care a maximum of 1 week prior to commencing anti‐EGFR treatment.

NLR was calculated using absolute neutrophil count/absolute lymphocyte count. PLR was calculated using absolute platelet count/absolute lymphocyte count. SII was calculated using (absolute neutrophil count × absolute platelet count)/absolute lymphocyte count.

Archival formalin‐fixed, paraffin‐embedded tumour tissue was analysed for AREG and EREG expression by IHC using previously developed artificial intelligence (AI) technologies [25].

### Statistical analysis

Baseline patient demographics and disease characteristics were recorded using descriptive statistics.

Participants were randomly partitioned into two equally sized training and validation sets using statistical software. A power calculation was performed to demonstrate sufficient power to perform this split. At a 5% significance level, using 50% of the population, there would be more than 80% power to detect a hazard ratio (HR) of 1.6 based on a 40% risk factor prevalence. With 100% of the population, there would be more than 80% power to detect a HR of 1.4 based on a 30% risk factor prevalence (Table S1). Training and validation sets were compared using Pearson's chi‐squared test.

Optimal cutoff values for NLR, PLR and SII were derived using receiver operating characteristic (ROC) curves and by calculating the Youden Index [26]. This method of determining biomarker cutoffs is useful as it obtains clinically usable dichotomous measures that maximise sensitivity and specificity, with equal weighting placed on each. As OS was the primary endpoint, the cutoff values calculated here were used after rounding up to a clinically interpretable value. Cutoff values identified in the training set were then tested in the validation set. Survival curves were generated using the Kaplan–Meier method. Categorical variables were evaluated in univariable Cox proportional hazards models and HRs and 95% confidence intervals (CIs) were estimated. The proportional hazards assumption was tested using Schoenfeld residuals. Odds ratios (ORs) and 95% CIs were estimated from logistic regression for ORR and DCR.

It was decided a priori that, should the prognostic value of the dichotomised measures be similar in both the training and validation sets, the two cohorts would be combined to investigate whether any associations between the superior and inferior prognostic groups and other patient or tumour characteristics might partly or wholly explain the association. In this situation, it was planned that multivariable Cox proportional hazards models would then be performed in the complete dataset for NLR, PLR and SII, adjusting for baseline characteristics that were prognostic in the univariable analysis.

Spearman's rank correlation coefficients were performed to assess correlations between NLR and PLR with AREG and EREG. Continuous AREG, continuous EREG and the combined ligand dichotomous measure (AREG or EREG > 20% vs. AREG and EREG ≤ 20%) were then assessed in multivariable models.

A *p*‐value less than 0.05 was considered statistically significant. Statistical analyses were performed using STATA, version 18 (StataCorp, Texas, USA).

## RESULTS

### Patient characteristics

**FIGURE 1:**
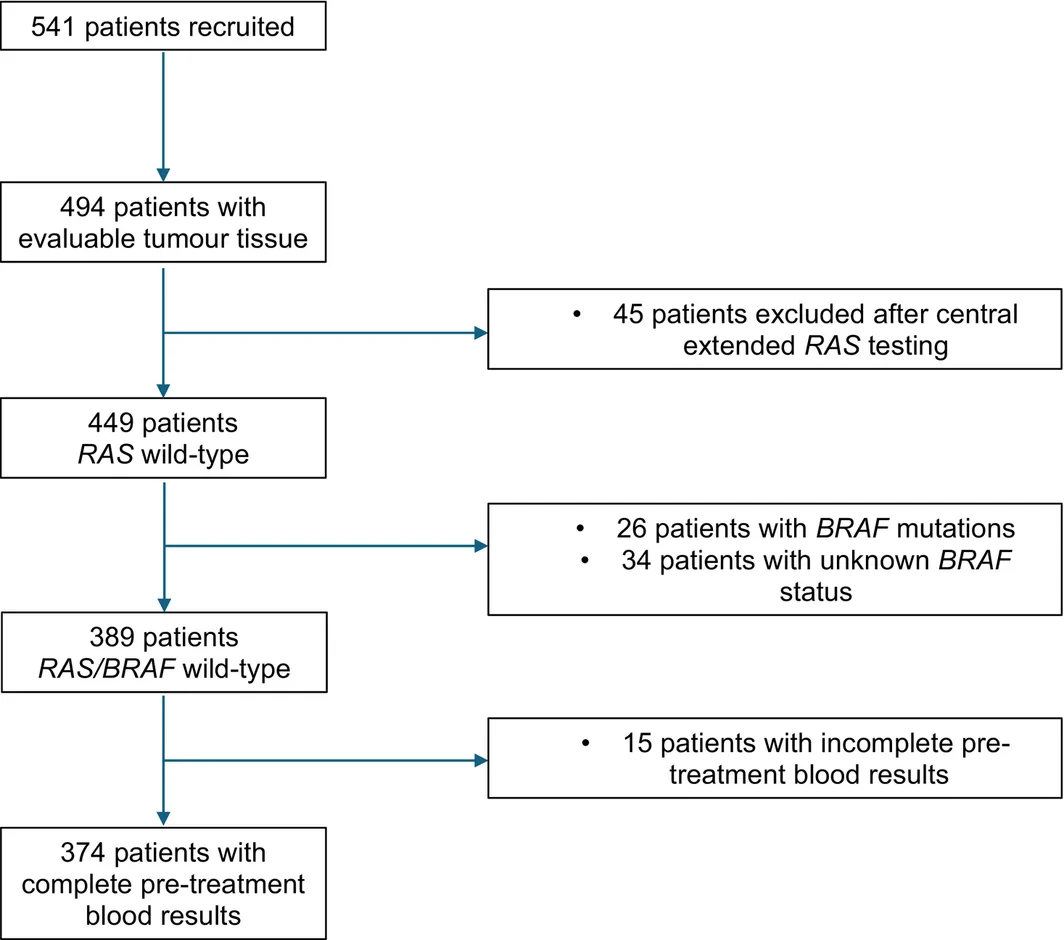
Consort flow diagram.

449 (83%) patients in the original patient cohort were *RAS* wild‐type following central extended *RAS* testing. 60 patients were excluded from the analysis presented here based on their *BRAF* mutation status at codon 600. A further 15 patients were excluded due to incomplete pre‐treatment blood results being available for the study, leaving 374 eligible patients (Figure 1).

Median follow‐up was 13.2 months. The mean age at starting treatment was 60.3 years. Males were predominant (67.1%). 90.9% of patients received anti‐EGFR therapy in combination with chemotherapy (67.7% received FOLFIRI, 19.5% received FOLFOX, 3.7% received other). Of the two licensed anti‐EGFR agents, almost two‐thirds of patients received cetuximab (65.2%) and one‐third received panitumumab (34.8%). 78.6% had a left‐primary tumour location (including rectum) (Table S2).

### Optimal cut points

Baseline characteristics were balanced between training and validation sets (Table S3).

The median NLR was 4 (IQR 2, 6). The internally derived cutoff value for NLR was 4, and so this was selected. The median PLR was 201 (IQR 150, 305). The internally derived cutoff value for PLR was 193 and so a pragmatic cutoff value of 200 was selected. NLR and PLR were strongly correlated (Spearman's rho = 0.75, *p* < 0.0001). We proceeded to examine the utility of combining information from each measure using SII ([absolute neutrophil count × absolute platelet count]/absolute lymphocyte count) to refine the prognostic effect. The median SII was 1093 (IQR 624, 1867). The internally derived cutoff value for SII was 874, and so a pragmatic cutoff value of 900 was selected (Figure S1).

### Survival outcomes

In the training set, high NLR, PLR and SII were all associated with a significantly inferior OS (Figure S2).

**FIGURE 2:**
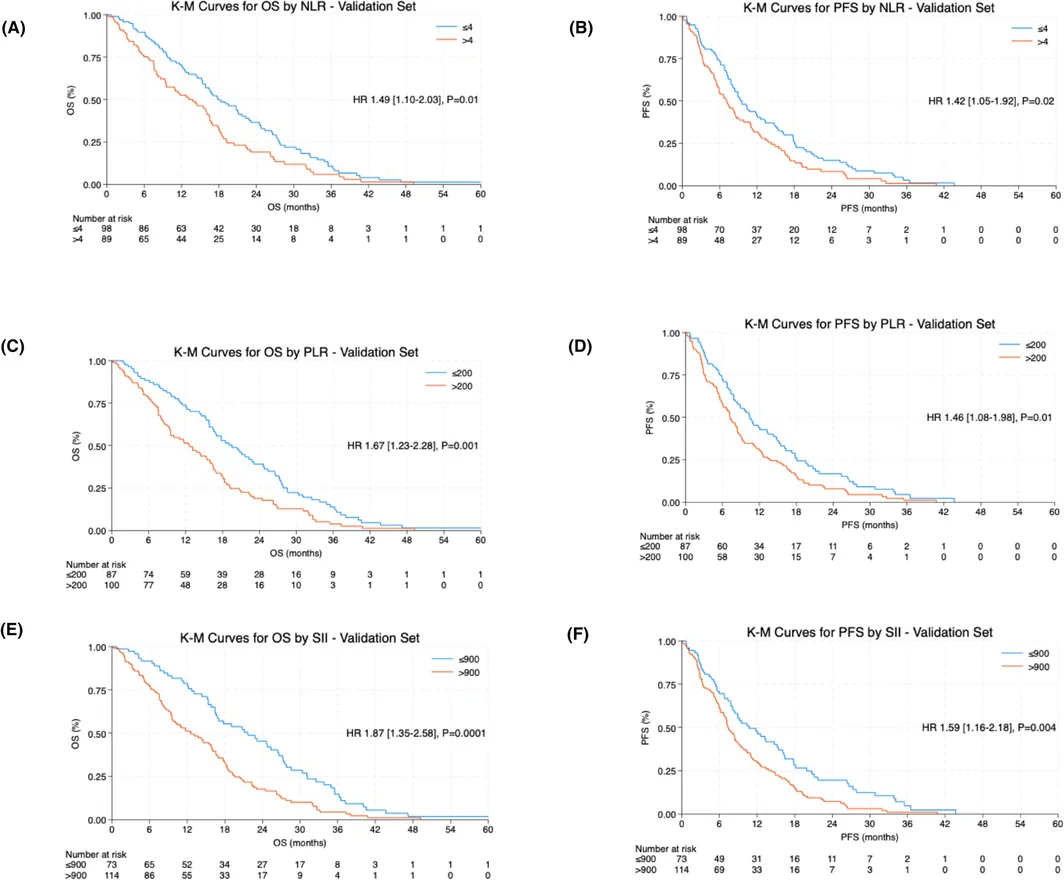
Kaplan‐Meier (K‐M) curves for overall survival (OS) and progression‐free survival (PFS) by (A, B) neutrophil‐to‐lymphocyte ratio (NLR), (C, D) platelet‐to‐lymphocyte ratio (PLR) and (E, F) systemic immune‐inflammation index (SII) in the validation set (*N* = 187).

These results were closely replicated in the validation set: high NLR, PLR and SII were all associated with a significantly inferior OS (NLR: median 16.1 vs. 11.7 months, unadjusted HR 1.49 [1.10–2.03], *p* = 0.01; PLR: median 16.8 vs. 11.4 months, unadjusted HR 1.67 [1.23–2.28], *p* = 0.001; SII: median 17.0 vs. 11.4 months, unadjusted HR 1.87 [1.35–2.58], *p* = 0.0001). Furthermore, high NLR, PLR and SII were all associated with a significantly inferior PFS (NLR: median 9.1 vs. 7.0 months, unadjusted HR 1.42 [1.05–1.92], *p* = 0.02; PLR: median 9.9 vs. 7.2 months, unadjusted HR 1.46 [1.08–1.09], *p* = 0.01; SII: median 9.6 vs. 7.3 months, unadjusted HR 1.59 [1.16–2.18], *p* = 0.004) (Figure 2).

Univariable analysis demonstrated that six baseline characteristics – performance status (1/2/3 vs. 0), chemotherapy backbone (none vs. FOLFIRI), primary tumour location (right colon vs. left colon/rectum), primary tumour removal (in situ vs. excised), line of treatment (second line or later vs. first line) and tumour differentiation grade (moderate/well vs. poor)—were significantly associated with OS (Table S4).

High NLR, PLR and SII all remained associated with a significantly inferior OS and PFS in multivariable analyses (Table 1).

**TABLE 1:** Multivariable analyses for overall survival (OS), progression‐free survival (PFS), objective response rate (ORR) and disease control rate (DCR) by neutrophil‐to‐lymphocyte ratio (NLR), platelet‐to‐lymphocyte ratio (PLR) and systemic immune‐inflammation index (SII).

Variable	Category	Complete set (*N* = 374)							
		OS		PFS		ORR		DCR	
		Adjusted HR (95% CI)	*p*	Adjusted HR (95% CI)	*p*	Adjusted OR (95% CI)	*p*	Adjusted OR (95% CI)	*p*
NLR	≤ 4	1.0		1.0		1.0		1.0	
(model 1)	> 4	1.29 (1.02–1.64)	**0.03**	1.44 (1.14–1.82)	**0.002**	0.49 (0.29–0.82)	**0.007**	0.70 (0.37–1.33)	0.28
PLR	≤ 200	1.0		1.0		1.0		1.0	
(model 2)	> 200	1.48 (1.18–1.86)	**0.0008**	1.69 (1.35–2.13)	**<0.0001**	0.65 (0.39–1.08)	0.10	0.33 (0.16–0.66)	**0.002**
SII	≤ 900	1.0		1.0		1.0		1.0	
(model 3)	> 900	1.63 (1.26–2.11)	**0.0002**	1.62 (1.26–2.08)	**0.0001**	0.63 (0.37–1.08)	0.09	0.66 (0.33–1.32)	0.24

*Note*: Each model adjusted for performance status, chemotherapy, primary tumour location, primary tumour removal, line of treatment and tumour differentiation grade.

### Efficacy outcomes

The optimal cut points identified for the ORR outcome were similar to those for the survival outcomes (data not shown). In univariable analyses, while effect sizes were similar, there was a significant improvement in ORR only for NLR (unadjusted OR 0.52 [0.28–0.97], *p* = 0.04), but not for PLR (unadjusted OR 0.74 [0.40–1.38], *p* = 0.35) or SII (unadjusted OR 0.57 [0.30–1.08], *p* = 0.08). No improvements were seen in DCR (NLR: unadjusted OR 0.75 [0.34–1.62], *p* = 0.46; PLR: unadjusted OR 0.53 [0.24–1.19], *p* = 0.12; SII: unadjusted OR 0.85 [0.38–1.88], *p* = 0.69). The improvement in ORR for NLR persisted in multivariable analyses (Table 1). To see if the improvement in ORR for NLR was driven by neutrophils or lymphocytes, we looked at individual counts and found that the improvement was mainly mediated by a lymphocyte effect rather than a neutrophil effect (continuous log_2_ neutrophils: unadjusted OR 0.82 [0.48–1.38], *p* = 0.45 and continuous log_2_ lymphocytes: unadjusted OR 2.16 [0.85–5.46], *p* = 0.11).

### Association with amphiregulin and epiregulin

It has previously been shown that high tumour AREG/EREG expression is associated with a significantly longer OS and PFS within this dataset [25], and the predictive effect of these ligands on anti‐EGFR efficacy has been confirmed in randomised datasets [27]. Here, there were no significant correlations between NLR and AREG or EREG (AREG: Spearman's rho = 0.08, *p* = 0.14; EREG: Spearman's rho = 0.05, *p* = 0.32) nor between PLR and AREG or EREG (AREG: Spearman's rho = 0.03, *p* = 0.53; EREG: Spearman's rho = 0.06, *p* = 0.25). When AREG and EREG were added to the multivariable survival models, as either continuous measures or as a combined dichotomised measure (AREG or EREG > 20% vs. AREG and EREG ≤ 20%), NLR and PLR remained independently prognostic for OS and PFS (Table 2).

**TABLE 2:** Multivariable analyses for overall survival (OS) and progression‐free survival (PFS) by neutrophil‐to‐lymphocyte ratio (NLR) and platelet‐to‐lymphocyte ratio (PLR).

Variable	Category	Complete set (N = 374)			
		OS		PFS	
		Adjusted HR (95% CI)	*p*	Adjusted HR (95% CI)	*p*
**Continuous AREG**					
NLR	≤ 4	1.0		1.0	
(model 1)	> 4	1.31 (1.03–1.66)	**0.03**	1.44 (1.14–1.81)	**0.002**
PLR	≤ 200	1.0		1.0	
(model 2)	> 200	1.49 (1.18–1.87)	**0.0007**	1.68 (1.34–2.11)	**< 0.0001**
**Continuous EREG**					
NLR	≤ 4	1.0		1.0	
(model 1)	> 4	1.31 (1.03–1.66)	**0.03**	1.45 (1.15–1.83)	**0.002**
PLR	≤ 200	1.0		1.0	
(model 2)	> 200	1.56 (1.23–1.97)	**0.0002**	1.75 (1.39–2.21)	**< 0.0001**
**Ligand dichotomous measure**					
NLR	≤ 4	1.0		1.0	
(model 1)	> 4	1.30 (1.03–1.65)	**0.03**		**0.003**
PLR	≤ 200	1.0		1.0	**0.003**
(model 2)	> 200	1.50 (1.19–1.88)	**0.0006**	1.68 (1.34–2.11)	**0.003**

*Note*: Each model additionally adjusted for amphiregulin (AREG) and epiregulin (EREG).

## DISCUSSION

We have shown that higher NLR and PLR are associated with an inferior prognosis in patients with *RAS/BRAF* wild‐type mCRC receiving anti‐EGFR therapy. Moreover, combining information from both measures in the SII resulted in improved prognostic discrimination.

Cutoff values obtained here using the ROC curve/Youden index method were similar to the median values. The NLR cutoff value of 4 and PLR cutoff value of 200 identified here are robust and supported across people with different cancers receiving different treatments—NLR of 4 being the optimal value from over 40,000 patients and PLR of 150 to 300 from nearly 13,000 patients [20, 21]. Two earlier small studies have examined the prognostic effect of NLR, PLR and SII in *RAS* wild‐type mCRC patients receiving cetuximab. These have shown NLR to be prognostic for both OS and PFS, but not PLR or SII [28, 29]. Crucially, they did not account for *BRAF* mutation status, yet we now know that *BRAF* mutations have a stronger negative prognostic effect than *RAS* mutations in the metastatic setting where deficient mismatch repair/microsatellite instability cancers are infrequently observed [30]. In this study, PLR appeared to have a stronger prognostic effect than NLR—an observation that was consistent across the training and validation sets. One explanation for this could be that PLR shows a T‐stage‐dependent increase [31], so a higher PLR is seen with deeper, more invasive tumours.

Earlier we described the different mechanisms underpinning the role of neutrophils, lymphocytes and platelets in cancer biology. These differences suggest how NLR and PLR might be prognostic for different reasons and justify looking at a combined score that might explain the enhanced effect. A recent meta‐analysis assessed the prognostic significance of SII in CRC. However, the obtained cutoff value of 550 was lower than our 900, emphasising the need for prognostic biomarkers to be examined in specific patient populations to gain wider traction in clinical practice. Again, *BRAF* mutation status was not described, and so this could account for the wide difference in cutoff values. Nevertheless, high SII was associated with a poorer OS (HR 1.78 [1.40–2.26], *p* < 0.001) [32]. The strength of the prognostic effect seen within the specific population described here, compared to that seen in a broader, less specific population, therefore gives greater confidence to clinicians about the implications of higher markers of systemic inflammation for their patients.

Only NLR was significantly associated with ORR. As ORR reflects treatment response rather than intrinsic prognosis, this suggests NLR might also have a predictive role. However, as there was no comparator group in this study, we can only directly infer a prognostic effect and cannot directly determine any predictive effect, which would need to be tested in a randomised clinical trial. PLR was not significantly associated with DCR in univariable analysis, though it reached significance in multivariable analysis, likely due to the smaller sample size in the univariable analyses. The effect of NLR on ORR appeared to be driven primarily by lymphocytes. Anti‐EGFR monoclonal antibodies, particularly cetuximab, are thought to mediate antibody‐dependent cellular cytotoxicity by tumour‐infiltrating CD8+ T cells [33]. Low lymphocytes may therefore impair these immune responses. This is supported by evidence that a pronounced lymphocytic response at the tumour invasive margin is associated with a lower NLR and improved prognosis in CRC [34].

Earlier work on the PICCOLO trial cohort (second‐line irinotecan with or without panitumumab in advanced CRC) [27] showed that AREG and EREG are predictive biomarkers of anti‐EGFR efficacy [23, 24]. Here we have observed that AREG/EREG expression does not correlate with markers of systemic inflammation. Although AREG has been shown to enhance intratumoural regulatory T cell function and promote immunosuppression [35], the biological mechanisms underlying neutrophilia, lymphocytopenia, and thrombocytosis in CRC are likely separate.

Utilising routine clinical parameter data provides a quick, inexpensive test using readily available data. We have demonstrated in this cohort that the average age of patients who receive SACT is lower than the average age of patients diagnosed with CRC. These markers might therefore be helpful in identifying older adults with a favourable prognosis who might still benefit from SACT, although these should not be used as a sole determinant of a patient's suitability for treatment. Information from prognostic biomarkers can also identify patients who are likely to have poorer survival outcomes, which can support patient discussions pertaining to how they wish to prioritise their time and with what quality. Markers of systemic inflammation have also shown promise in helping select appropriate patients for rechallenge therapy [36], as well as liver resection for CRC metastasis [37].

The main strength of this study is that it uses real‐world data and is generalisable given the multi‐centre approach. Splitting a single retrospective cohort into training and validation sets is a recognised method to improve validity of findings, and there is precedent for this [38, 39]. However, this approach can also reduce power. Here we were able to demonstrate that we could still maintain sufficient power and our findings remained statistically significant, emphasising the strength of the prognostic effect. The main limitation of this study is that it requires external validation. Finally, response assessments were investigator‐defined and were not reported according to RECIST criteria—although the reproducibility of the findings in the training and validation sets suggests this could in fact be viewed as enhancing the generalisability of the study findings to routine clinical practice.

## CONCLUSION

In conclusion, this study demonstrates that NLR, PLR and SII are useful prognostic indicators for patients with *RAS/*BRAF wild‐type mCRC where treatment using anti‐EGFR agents is proposed. NLR and PLR are independently prognostic, and combining information from both in SII enhances prognostic discrimination. The prognostic effect of these markers was independent of AREG/EREG—two mechanistic predictive biomarkers for anti‐EGFR efficacy—suggesting there are differing underlying biological mechanisms.

## AUTHOR CONTRIBUTIONS

**Jordan W. Appleyard:** Conceptualization; data curation; formal analysis; investigation; methodology; project administration; writing – original draft; writing – review and editing. **Faye Elliott:** Formal analysis; methodology; writing – review and editing. **Nancy L. Sapanara:** Investigation. **Liping Zhang:** Investigation. **Uday Kurkure:** Software. **Jenny F. Seligmann:** Methodology; supervision; writing – review and editing. **Nicholas P. West:** Methodology; supervision; writing – review and editing. **Kandavel Shanmugam:** Funding acquisition; project administration. **Philip Quirke:** Methodology; supervision; writing – review and editing. **Christopher J. M. Williams:** Conceptualization; data curation; supervision; investigation; methodology; project administration; writing – review and editing.

## FUNDING INFORMATION

The work was supported by Yorkshire Cancer Research (L386, L394); National Institute for Health and Care Research, Leeds Biomedical Research Centre (NIHR203331); Cancer Research UK (RCCCTF‐Nov21/100001); UK Research and Innovation (UKRI) (104687).

## CONFLICT OF INTEREST STATEMENT

NLS, LZ, UK and KS are employees of Roche Diagnostics Solutions and share ownership in Roche Holding AG.

JFS reports: Advisory/Consultancy: BMS, Johnstone & Johnstone, Merck Serono, GSK, Nanobiotix, Nordic Pharmaceuticals, Sanofi, Servier, Takeda. Research Funding: Amgen, GSK, Pierre Fabre Medicament, Merck Serono, Roche Diagnostics. Speaker Fees: GSK, Merck Serono, Pierre Fabre Medicament, Servier, Takeda. Travel: Takeda.

NPW reports: Advisory/Consultancy: Bristol Myers Squibb, Astellas, Pfizer, Amgen, GSK, Servier. Research Funding: Roche Diagnostics, Pierre Fabre, GSK, Amgen.

PQ reports: Advisory/Consultancy: Roche Diagnostics. Research Funding: Roche Diagnostics. Speaker Fees: Roche Diagnostics.

CJMW reports: Advisory/Consultancy: Boehringer Ingelheim, MJH Life Sciences. Speaker Fees: Incyte, GSK, Servier, Merck Serono, Roche Diagnostics, Tactics MD. Travel: IPSEN.

JWA and FE declare no conflict of interest.

## Supporting information


**Table S1.** Power calculations for overall survival based on various‐sized populations.
**Table S2.** Baseline patient demographics and disease characteristics (*N* = 374).
**Table S3.** Comparison between training and validation sets.
**Figure S1.** Receiver operating characteristic (ROC) curves for overall survival by (a) neutrophil‐to‐lymphocyte ratio (NLR), (b) platelet‐to‐lymphocyte ratio (PLR) and (c) systemic immune‐inflammation index (SII).
**Figure S2.** Kaplan–Meier (K‐M) curves for overall survival (OS) by (a) neutrophil‐to‐lymphocyte ratio (NLR), (b) platelet‐to‐lymphocyte ratio (PLR) and (c) systemic immune‐inflammation index (SII) in the training set (*N* = 187).
**Table S4.** Univariable analysis for overall survival (OS) in the validation set (*N* = 187).

## Data Availability

The data generated in this study are available upon request from the corresponding author.
