# Supplementary material for: Systemic inflammation and prognosis in patients with RAS / BRAF wild‐type metastatic colorectal cancer receiving anti‐EGFR therapy: A real‐world cohort study
Source: Colorectal Dis. 2026 Sep 25;28(10):e70639. doi: 10.1111/codi.70639 (PMC13615331; doi:10.1111/codi.70639)
Supplement: Supplementary file 1 [file CODI-28-0-s001.docx]

## Supplementary material

| **Numbers based on 100% of the population:** | | | |  |  |  |  |
| --- | --- | --- | --- | --- | --- | --- | --- |
| N=374, n=331 OS events, n=349 PFS events | | | |  |  |  |  |
|  |  |  |  |  |  |  |  |
|  | **HR** | | | | |  |  |
| **Prevalence of risk factor** | **1.2** | **1.4** | **1.5** | **1.6** | **1.8** |  |  |
| **0.2** | 26 | 69 | **84** | **93** | **99** |  |  |
| **0.3** | 33 | **80** | **92** | **97** | **99** |  |  |
| **0.4** | 37 | **85** | **95** | **99** | **99** |  |  |
| **0.5** | 38 | **86** | **96** | **99** | **99** |  |  |
| **0.6** | 37 | **85** | **95** | **99** | **99** |  |  |
|  |  |  |  |  |  |  |  |
| **Numbers based on 70% of the population:** | | | |  |  |  |  |
| N=262, n=232 OS events, n=244 PFS events | | | |  |  |  |  |
|  |  |  |  |  |  |  |  |
|  | **HR** | | | | |  |  |
| **Prevalence of risk factor** | **1.2** | **1.4** | **1.5** | **1.6** | **1.8** |  |  |
| **0.2** | 20 | 54 | 70 | **82** | **95** |  |  |
| **0.3** | 25 | 65 | **81** | **91** | **98** |  |  |
| **0.4** | 27 | 71 | **86** | **94** | **99** |  |  |
| **0.5** | 28 | 73 | **87** | **95** | **99** |  |  |
| **0.6** | 27 | 71 | **86** | **94** | **99** |  |  |
|  |  |  |  |  |  |  |  |
| **Numbers based on 50% of the population:** | | | |  |  |  |  |
| N=187, n=166 OS events, n=175 PFS events | | | |  |  |  |  |
|  |  |  |  |  |  |  |  |
|  | **HR** | | | | |  |  |
| **Prevalence of risk factor** | **1.2** | **1.4** | **1.5** | **1.6** | **1.8** |  |  |
| **0.2** | 15 | 41 | 55 | 68 | **86** |  |  |
| **0.3** | 19 | 51 | 67 | 79 | **93** |  |  |
| **0.4** | 21 | 57 | 73 | **84** | **96** |  |  |
| **0.5** | 22 | 58 | 74 | **86** | **97** |  |  |
| **0.6** | 21 | 57 | 73 | **84** | **96** |  |  |
|  |  |  |  |  |  |  |  |
| **Numbers based on 30% of the population:** | | | |  |  |  |  |
| N=112, n=99 OS events, n=105 PFS events | | |  |  |  |  |  |
|  |  |  |  |  |  |  |  |
|  | **HR** | | | | |  |  |
| **Prevalence of risk factor** | **1.2** | **1.4** | **1.5** | **1.6** | **1.8** |  |  |
| **0.2** | 11 | 27 | 36 | 46 | 65 |  |  |
| **0.3** | 13 | 34 | 46 | 57 | 76 |  |  |
| **0.4** | 14 | 37 | 51 | 63 | **82** |  |  |
| **0.5** | 15 | 39 | 52 | 65 | **83** |  |  |
| **0.6** | 14 | 37 | 51 | 63 | **82** |  |  |
|  |  |  |  |  |  |  |  |
| **Supplementary Table 1:** Power calculations for overall survival based on various sized  populations. | | | | | | | |
| Power (%) using 5% significance level. | | |  |  |  |  |  |

|  |  | **N (%)** |  |
| --- | --- | --- | --- |
| **Variable** | **Category** | **Unless otherwise stated** |  |
| Age at starting treatment (years) |  | Mean 60.3 (SD 10.8) |  |
|  | ≤60 | 179 (47.9) |  |
|  | >60 | 195 (52.1) |  |
| Sex | Male | 251 (67.1) |  |
|  | Female | 123 (32.9) |  |
| Performance status | 0 | 149 (39.8) |  |
|  | 1 | 119 (31.8) |  |
|  | 2 or 3 | 27 (7.2) |  |
|  | Unknown | 79 (21.1) |  |
| Chemotherapy | FOLFIRI | 253 (67.7) |  |
|  | FOLFOX | 73 (19.5) |  |
|  | Other | 14 (3.7) |  |
|  | None | 34 (9.1) |  |
| Anti-EGFR agent | Cetuximab | 244 (65.2) |  |
|  | Panitumumab | 130 (34.8) |  |
| Primary tumour location | Left colon/Rectum | 294 (78.6) |  |
|  | Right colon | 80 (21.4) |  |
| Primary tumour removal | Excised | 212 (56.7) |  |
|  | *In situ* | 162 (43.3) |  |
| Previous neoadjuvant radiotherapy | No | 86 (23.0) |  |
| (rectal cancer only) | Yes | 40 (10.7) |  |
|  | Not applicable | 248 (66.3) |  |
| Previous adjuvant chemotherapy | No | 246 (65.8) |  |
|  | Yes | 128 (34.2) |  |
| Line of treatment | First line | 314 (84.0) |  |
|  | Second line or later | 60 (16.0) |  |
| Tumour differentiation grade | Poor | 49 (13.1) |  |
|  | Moderate/well | 265 (70.9) |  |
|  | Unknown | 60 (16.0) |  |
| Local recurrence | No | 173 (46.3) |  |
|  | Yes | 37 (9.9) |  |
|  | Not applicable | 162 (43.3) |  |
|  | Unknown | 2 (0.5) |  |
| Number of metastatic sites grouped | 0 or 1 | 138 (36.9) |  |
|  | 2 or more | 228 (61.0) |  |
|  | Unknown | 8 (2.1) |  |
| Overall survival event | Censored | 43 (11.5) |  |
|  | Died | 331 (88.5) |  |
| Progression-free survival event | Censored | 25 (6.7) |  |
|  | Progressed/died | 349 (93.3) |  |
| Response rate | SD/PD | 152 (40.6) |  |
|  | CR/PR | 177 (47.3) |  |
|  | No scan | 45 (12.0) |  |
| Disease control rate | PD | 64 (17.1) |  |
|  | SD/PR/CR | 265 (70.9) |  |
|  | No scan | 45 (12.0) |  |
| Continuous AREG |  | Mean 55.0 (SD 32.1) |  |
| Continuous EREG |  | Mean 52.6 (SD 35.2) |  |
| Ligand dichotomous measure | AREG and EREG ≤20% | 63 (16.8) |  |
|  | AREG or EREG >20% | 311 (83.2) |  |
|  |  |  |  |
| **Supplementary Table 2:** Baseline patient demographics and disease characteristics (N=374). | | | |

|  |  | **Training (N=187)** | **Validation (N=187)** |  |
| --- | --- | --- | --- | --- |
| **Variable** | **Category** | **N (%)** | **N (%)** | **P value** |
| Age at starting treatment (years) | ≤60 | 88 (47.1) | 91 (48.7) |  |
|  | >60 | 99 (52.9) | 96 (51.3) | 0.76 |
| Sex | Male | 125 (66.8) | 126 (67.4) |  |
|  | Female | 62 (33.2) | 61 (32.6) | 0.91 |
| Performance status | 0 | 83 (44.4) | 66 (35.3) |  |
|  | 1 | 52 (27.8) | 67 (35.8) |  |
|  | 2 or 3 | 12 (6.4) | 15 (8.0) |  |
|  | Unknown | 40 (21.4) | 39 (20.9) | 0.24 |
| Chemotherapy | FOLFIRI | 127 (67.9) | 126 (67.4) |  |
|  | FOLFOX | 38 (20.3) | 35 (18.7) |  |
|  | Other | 9 (4.8) | 5 (2.7) |  |
|  | None | 13 (7.0) | 21 (11.2) | 0.37 |
| Anti-EGFR agent | Cetuximab | 118 (63.1) | 126 (67.4) |  |
|  | Panitumumab | 69 (36.9) | 61 (32.6) | 0.39 |
| Primary tumour location | Left colon/Rectum | 150 (80.2) | 144 (77.0) |  |
|  | Right colon | 37 (19.8) | 43 (23.0) | 0.45 |
| Primary tumour removal | Excised | 112 (59.9) | 100 (53.5) |  |
|  | *In situ* | 75 (40.1) | 87 (46.5) | 0.21 |
| Previous neoadjuvant radiotherapy | No | 45 (24.1) | 41 (21.9) |  |
| (rectal cancer only) | Yes | 19 (10.2) | 21 (11.2) |  |
|  | Not applicable | 123 (65.8) | 125 (66.8) | 0.86 |
| Previous adjuvant chemotherapy | No | 121 (64.7) | 125 (66.8) |  |
|  | Yes | 66 (35.3) | 62 (33.2) | 0.66 |
| Line of treatment | First line | 160 (85.6) | 154 (82.3) |  |
|  | Second line or later | 27 (14.4) | 33 (17.7) | 0.40 |
| Tumour differentiation grade | Poor | 25 (13.4) | 24 (12.8) |  |
|  | Moderate/well | 132 (70.6) | 133 (71.1) |  |
|  | Unknown | 30 (16.0) | 30 (16.0) | 0.99 |
| Local recurrence | No | 90 (48.1) | 83 (44.4) |  |
|  | Yes | 20 (10.7) | 17 (9.1) |  |
|  | Not applicable | 75 (40.1) | 87 (46.5) |  |
|  | Unknown | 2 (1.1) | 0 (0.0) | 0.33 |
| Number of metastatic sites grouped | 0 or 1 | 71 (38.0) | 67 (35.8) |  |
|  | 2 or more | 112 (59.9) | 116 (62.0) |  |
|  | Unknown | 4 (2.1) | 4 (2.1) | 0.91 |
|  |  |  |  |  |
| **Supplementary Table 3:** Comparison between training and validation sets. | | |  |  |

| **a.** |  |  |  |  |  |  |
| --- | --- | --- | --- | --- | --- | --- |
|  |  |  |  |  |  |  |
|  |  |  |  |  |  |  |
|  |  |  |  |  |  |  |
|  |  |  |  |  |  |  |
|  |  |  |  |  |  |  |
|  |  |  |  |  |  |  |
|  |  |  |  |  |  |  |
|  |  |  |  |  |  |  |
|  |  |  |  |  |  |  |
|  |  |  |  |  |  |  |
|  |  |  |  |  |  |  |
| **b.** |  | | | | |  |
|  |  |  |  |  |  |  |
|  |  |  |  |  |  |  |
|  |  |  |  |  |  |  |
|  |  |  |  |  |  |  |
|  |  |  |  |  |  |  |
|  |  |  |  |  |  |  |
|  |  |  |  |  |  |  |
|  |  |  |  |  |  |  |
|  |  |  |  |  |  |  |
|  |  |  |  |  |  |  |
|  |  |  |  |  |  |  |
| **c.** |  | | | | |  |
|  |  |  |  |  |  |  |
|  |  |  |  |  |  |  |
|  |  |  |  |  |  |  |
|  |  |  |  |  |  |  |
|  |  |  |  |  |  |  |
|  |  |  |  |  |  |  |
|  |  |  |  |  |  |  |
|  |  |  |  |  |  |  |
|  |  |  |  |  |  |  |
|  |  |  |  |  |  |  |
|  |  |  |  |  |  |  |
| **Supplementary figure 1:** Receiver Operating Characteristic (ROC) curves for overall survival by a) neutrophil-to-lymphocyte ratio | | | | | |  |
| (NLR), b) platelet-to-lymphocyte ratio (PLR) and | | | | | | |
| c) systemic immune-inflammation index (SII). | | | | |  |  |

| **a.** |  |  |  |  |  |  |  |
| --- | --- | --- | --- | --- | --- | --- | --- |
|  |  |  |  |  |  |  |  |
|  |  |  |  |  |  |  |  |
|  |  |  |  |  |  |  |  |
|  |  |  |  |  |  |  |  |
|  |  |  |  |  |  |  |  |
|  |  |  |  |  |  |  |  |
|  |  |  |  |  |  |  |  |
|  |  |  |  |  |  |  |  |
|  |  |  |  |  |  |  |  |
|  |  |  |  |  |  |  |  |
|  |  |  |  |  |  |  |  |
| **b.** |  | | | | |  |  |
|  |  |  |  |  |  |  |  |
|  |  |  |  |  |  |  |  |
|  |  |  |  |  |  |  |  |
|  |  |  |  |  |  |  |  |
|  |  |  |  |  |  |  |  |
|  |  |  |  |  |  |  |  |
|  |  |  |  |  |  |  |  |
|  |  |  |  |  |  |  |  |
|  |  |  |  |  |  |  |  |
|  |  |  |  |  |  |  |  |
|  |  |  |  |  |  |  |  |
| **c.** |  | | | | |  |  |
|  |  |  |  |  |  |  |  |
|  |  |  |  |  |  |  |  |
|  |  |  |  |  |  |  |  |
|  |  |  |  |  |  |  |  |
|  |  |  |  |  |  |  |  |
|  |  |  |  |  |  |  |  |
|  |  |  |  |  |  |  |  |
|  |  |  |  |  |  |  |  |
|  |  |  |  |  |  |  |  |
|  |  |  |  |  |  |  |  |
|  |  |  |  |  |  |  |  |
| **Supplementary Figure 2:** Kaplan-Meier (K-M) curves for overall survival (OS) by | | | | | | | |
| a) neutrophil-to-lymphocyte ratio (NLR), b) platelet-to-lymphocyte ratio (PLR) | | | | | | |  |
| and c) systemic immune-inflammation index (SII) in the training set (N=187). | | | | | | |  |

|  |  | **OS** |  |  |
| --- | --- | --- | --- | --- |
| **Variable** | **Category** | **Overall/died** | **Unadjusted HR (95% CI)** | **P value** |
| Age at starting treatment (years) | ≤60 | 83/91 | 1.0 |  |
|  | >60 | 82/96 | 0.74 (0.55-1.01) | 0.06 |
| Sex | Male | 109/126 | 1.0 |  |
|  | Female | 56/61 | 1.31 (0.95-1.81) | 0.11 |
| Performance status * | 0 | 54/66 | 1.0 |  |
|  | 1 | 57/67 | 1.52 (1.03-2.24) | **0.03** |
|  | 2 or 3 | 15/15 | 4.21 (2.28-7.78) | **<0.0001** |
| Chemotherapy | FOLFIRI | 109/126 | 1.0 |  |
|  | FOLFOX | 31/35 | 1.27 (0.85-1.90) | 0.24 |
|  | Other | 4/5 | 1.12 (0.41-3.06) | 0.82 |
|  | None | 21/21 | 3.94 (2.40-6.46) | **<0.0001** |
| Anti-EGFR agent | Cetuximab | 113/126 | 1.0 |  |
|  | Panitumumab | 52/61 | 1.19 (0.85-1.66) | 0.30 |
| Primary tumour location | Left colon/Rectum | 124/144 | 1.0 |  |
|  | Right colon | 41/43 | 1.63 (1.14-2.34) | **0.008** |
| Primary tumour removal | Excised | 86/100 | 1.0 |  |
|  | *In situ* | 79/87 | 1.48 (1.08-2.03) | **0.01** |
| Previous neoadjuvant radiotherapy | No | 36/41 | 1.0 |  |
| (rectal cancer only) * | Yes | 19/21 | 1.11 (0.63-1.96) | 0.72 |
| Previous adjuvant chemotherapy | No | 110/125 | 1.0 |  |
|  | Yes | 55/62 | 0.89 (0.64-1.23) | 0.47 |
| Line of treatment | First line | 132/154 | 1.0 |  |
|  | Second line or later | 33/33 | 2.19 (1.49-3.23) | **<0.0001** |
| Tumour differentiation grade * | Poor | 23/24 | 1.0 |  |
|  | Moderate/well | 114/133 | 0.61 (0.38-0.95) | **0.03** |
| Local recurrence * | No | 72/83 | 1.0 |  |
|  | Yes | 14/17 | 1.39 (0.78-2.48) | 0.26 |
| Number of metastatic sites grouped * | 0 or 1 | 57/67 | 1.0 |  |
|  | 2 or more | 104/116 | 1.08 (0.78-1.50) | 0.63 |
|  |  |  |  |  |
| **Supplementary Table 4:** Univariable analysis for overall survival (OS) in the validation set (N=187)**.** | | | |  |
| * Test excludes unknowns/not applicable. | |  |  |  |
